# Prediction of Histopathological Grade of Hepatocellular Carcinoma by Gadoxetic Acid–Enhanced Magnetic Resonance Imaging Radiomics Features

**DOI:** 10.5152/tjg.2025.25431

**Published:** 2025-11-21

**Authors:** Bisar Akbas, Huseyin Tugsan Balli, Kairgeldy Aikimbaev, Ferhat Can Piskin, Kivilcim Eren Erdogan, Puren Sevinc Yucel

**Affiliations:** 1 Department of Radiology, Çukurova University School of Medicine, Adana, Türkiye; 2 Department of Pathology, Çukurova University School of Medicine, Adana, Türkiye; 3 Department of Biostatistics, Çukurova University School of Medicine, Adana, Türkiye

**Keywords:** Gadoxetic acid, Gd-EOB-DTPA, HCC, hepatocellular carcinoma, histopathological grade, hepatobiliary phase, radiomics

## Abstract

**Background/Aims:**

This study aimed to evaluate preoperative models based on gadoxetic acid (Gd-EOB-DTPA [gadolinium ethoxybenzyl diethylenetriamine pentaacetic acid])–enhanced magnetic resonance imaging (MRI) radiomics for predicting the histopathological grade of hepatocellular carcinoma (HCC).

**Materials and Methods:**

This retrospective study included 68 treatment-naïve patients with histopathologically confirmed HCC from September 2015 to November 2021. Tumors were categorized into well-differentiated and non-well-differentiated groups. Radiomics features were extracted from preoperative hepatobiliary phase MRI images. Logistic regression (LR) with least absolute shrinkage and selection operator selection was used to identify key radiomics features and clinical parameters. Three models—radiomics, clinical, and combined clinical-radiomics (CCR)—were developed to predict HCC differentiation.

**Results:**

The radiomics and clinical models achieved area under the curve (AUC) values of 0.803 and 0.749, respectively, while the CCR model showed superior performance (AUC 0.827). In the clinical model, the albumin-bilirubin score was an independent risk factor (*P* < .05). The radiomics score was significantly lower in well-differentiated tumors (*P* < .001). Radiomics features were independent predictors in the CCR model (*P* = .005).

**Conclusion:**

Radiomics features from hepatobiliary phase MRI and clinical parameters can effectively predict the differentiation grade of HCC, aiding in preoperative decision-making. However, the study is limited by its small sample size and the absence of external validation; therefore, further multicenter studies are necessary.

**Main Points**

- Gadoxetic acid–enhanced (Gd-EOB-DTPA) magnetic resonance imaging radiomics can non-invasively predict histopathological grade in hepatocellular carcinoma (HCC).
- A combined radiomics-clinical model improves prediction accuracy and may guide personalized treatment planning.
- These findings support the potential of radiomics as a valuable adjunct in clinical decision-making for individualized treatment planning in HCC patients.

## Introduction

Hepatocellular carcinoma (HCC), a significant cause of cancer-related mortality, highlights the critical need for enhanced diagnostic and treatment options.^1^ Despite the presence of various treatment options, early and late recurrence is common, leading to inadequate survival rates in HCC. Accurate assessment of the histopathological grade of HCC is crucial, as it directly influences clinical decisions regarding treatment strategies and patient management.^2^ Several studies have emphasized the relevance of poor tumor differentiation and its relationship with lower overall survival and high recurrence.^3^^,^^4^ Edmondson–Steiner (ES) grading system is used to evaluate the level of differentiation in HCC. Edmondson–Steiner grade significantly predicts treatment response, prognosis, and overall survival.^5^ Edmondson–Steiner grading can be determined through core needle biopsy or surgical tissue sampling. Therefore, evaluating the HCC grade on preoperative imaging may contribute to the treatment planning.

Radiomics is a fast-developing field that extracts various quantitative features from medical images. These features capture various aspects of the image, including texture, shape, intensity, and spatial relationships, which may provide insight into underlying tissue biology and guide clinical decision-making.^6^ By leveraging advanced applications and machine learning, radiomics has the potential to identify unique imaging biomarkers associated with histopathological features.^7^

Hepatocyte-specific contrast material gadoxetic acid, also known as Gd-EOB-DTPA (Primovist; Bayer), improves visualization of hepatic tissue and enhances lesion characterization.^8^ This agent is transported into the hepatocytes over organic anion transporting polypeptide 1B3 (OATP1B3) and is excreted into the bile ducts after 10-20 minutes.^9^ In the hepatobiliary phase, differences in signal properties are observed in HCC tumors according to different degrees of differentiation. Conventional studies have shown differences in signal characteristics in the gadoxetic acid–enhanced magnetic resonance imaging (MRI) hepatobiliary phase based on HCC grades.^10^^,^^11^ This contrast material can also evaluate liver structure and function, providing crucial information for non-invasive assessment of HCC grades in the hepatobiliary phase. Recent research indicates that radiomics on gadoxetic acid–enhanced MRI might stage liver fibrosis, liver failure, and cytokeratin 19 (CK19) expression in HCC and predict early and late recurrence.^12^^,^^13^ Gd-EOB-DTPA–enhanced MRI combined with radiomics analysis represents a promising non-invasive and reproducible approach to predict the histopathological grade of HCC. This study is one of the few in the literature that uses radiomics features of gadoxetic acid–enhanced MRI to estimate preoperative HCC histopathological grade.

This study aims to develop preoperative models to predict the ES grade of HCC by combining radiomics features from gadoxetic acid–enhanced MRI with clinical parameters. By using radiomics analysis, the hope is to create a non-invasive and reliable method for assessing the histopathological grade of HCC. This approach could help clinicians make better treatment decisions and ultimately improve patient outcomes.

## Materals and Methods

This single-center retrospective study received approval from the Institutional Clinical Research Ethics Committee of Çukurova University (no.: 84/12/21; date: December 8, 2021). Due to its retrospective nature, informed consent from patients was not required. The study was conducted in accordance with the Declaration of Helsinki.

### Study Population

**Figure 1.**
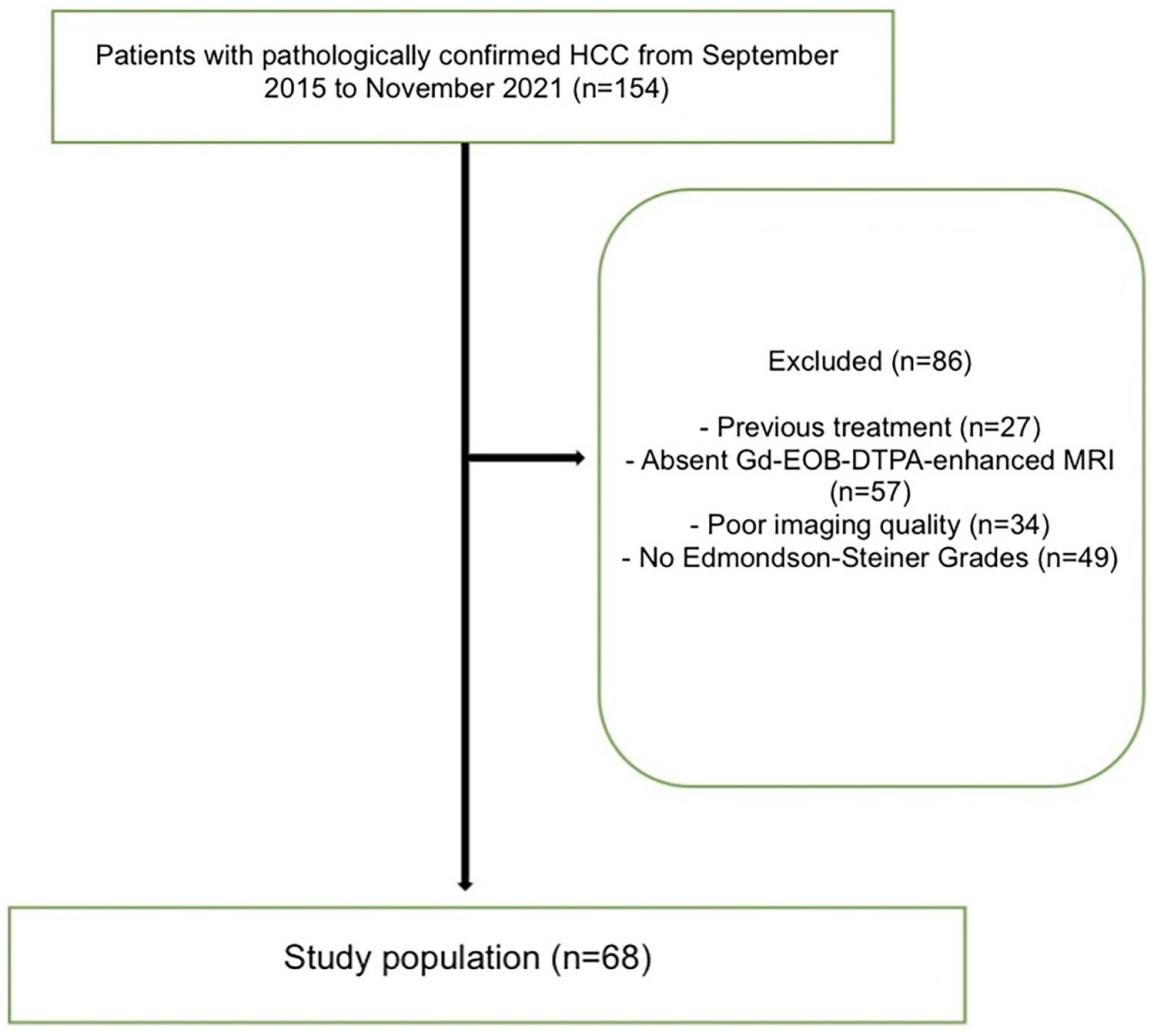
Flow chart of inclusion and exclusion criteria.

Patients diagnosed with HCC via Tru-cut biopsy between September 2015 and November 2021 were included in this retrospective study. The criteria for inclusion in this study were as follows: (i) Gd-EOB-DTPA–enhanced MRI before treatment; (ii) histological report according to ES grades; and (iii) no previous treatment, including systemic, endovascular, and percutaneous ablation. Exclusion criteria from the study were as follows: (i) poor quality of magnetic resonance images due to motion artifacts and (ii) inability to state tumor margins in infiltrative-type HCC. The flow diagram illustrating the inclusion and exclusion criteria for the study can be seen in Figure 1.

### Assessment of Histological Grading

A preoperative core needle biopsy collected tissue samples for histological examination. The diagnosis and grading of HCC were accurately determined using the established ES system. Tumor differentiation was classified as follows: well-differentiated (n = 33), moderately differentiated (n = 24), and poorly differentiated (n = 11). The differentiation groups were carefully defined as either well-differentiated or non-well-differentiated (moderately and poorly differentiated) to capture the spectrum of differentiation levels accurately.

### Magnetic Resonance Imaging Protocol

Magnetic resonance imaging was performed on 2 machines: 3.0-T (Ingenia, Philips Medical Systems, Best, The Netherlands) equipped with a 16-channel dStream Torso coil or 1.5-T General Electric HDI ECHOSPEED (GE Healthcare, Milwaukee, USA) featuring an 8-channel large phased array Torso coil. The dynamic contrast-enhanced phase MRI was obtained using the following specifications: TR (repetition time) = 3.5-4.5 ms (3.0-T) or 4.5-6.5 ms (1.5-T), TE (echo time) = 1.2-2.0 ms (3.0-T) or 2.0-2.4 ms (1.5-T), a flip angle of 10°-12° (3.0-T) or 12°-15° (1.5-T), a field of view of 350-400 mm (3.0-T) or 350-420 mm (1.5-T), a matrix size of 256 × 256 to 320 × 320 (3.0-T) or 192 × 256 to 256 × 256 (1.5-T), slice thickness = 3 mm; interslice gap = 0.6 mm. Dynamic images were captured after contrast was administered in the arterial phase (AP) (20 seconds), portal venous phase (50 seconds), equilibrium phase (90 seconds), and final hepatobiliary phase (HBP) (20 minutes) using a T1-weighted mDixon-3D-GRE or LAVA sequence. A power injector delivered a bolus of 0.1 mL/kg gadoxetic acid followed by an immediate 20 mL 0.9% saline flush via antecubital venous catheter (Spectris MR Injector System, Medrad, USA).

### Magnetic Resonance Imaging Radiomics Analysis

The images obtained with gadoxetic acid–enhanced MRI were analyzed by 1 radiologist blinded to the patient’s laboratory and clinicopathological information. The first step was to download hepatobiliary phase images (20 minutes) in the Digital Imaging and Communication in Medicine format with a 3.0 mm slice thickness from the picture archiving and communication system. Subsequently, the tumors were manually segmented utilizing regions-of-interest analysis software that received FDA (U.S. Food and Drug Administration) approval (Olea Sphere software, Olea Medical, La Ciotat, France) by a radiologist (5 years of experience) independently. During any conflicts, disagreements were resolved through consensus. Each patient had 111 separate radiomics features extracted, including first-order features (histogram features), second-order features, and shape features. Differences in signal-to-noise ratio and spatial resolution between 1.5T and 3.0T scanners were noted but harmonized via standardized feature extraction.

### Feature Selection and Establishment of the Models

The least absolute shrinkage and selection operator (LASSO) method, proposed by Tibshirani in 1996, is widely used for reducing and selecting variables in high-dimensional data, particularly in recent radiomics studies. Since the number of radiomics features extracted based on each patient’s images exceeded the number of patients, selecting radiomics features was implemented using LASSO. Combining the LASSO method with the logistic regression (LR) model enables the precise selection of vital radiomics features and clinical parameters. To select the best model and reduce overfitting, 5-fold cross-validation was implemented to optimize the parameter value that most accurately fits the model to the data. According to the determined tuning parameters, 7 radiomics features were selected, which are given in Table 1. Then, to determine each patient’s radiomics score (rad score), the linear combination of the selected features was calculated, and each value was multiplied by its respective coefficient. Based on the LR model, the clinical prediction model was established with the following variables: age, sex, alpha-fetoprotein, history of hepatitis B or C, ascites, tumor size, tumor number, tumor distribution, portal vein tumor thrombosis, portal hypertension, Child–Pugh score, albumin-bilirubin (ALBI) score, total bilirubin, alanine aminotransferase, neutrophil-to-lymphocyte ratio, and international normalized ratio. In addition, the combined radiomics-clinical model was built by integrating radiomics signatures and clinical parameters.

**Figure 2.**
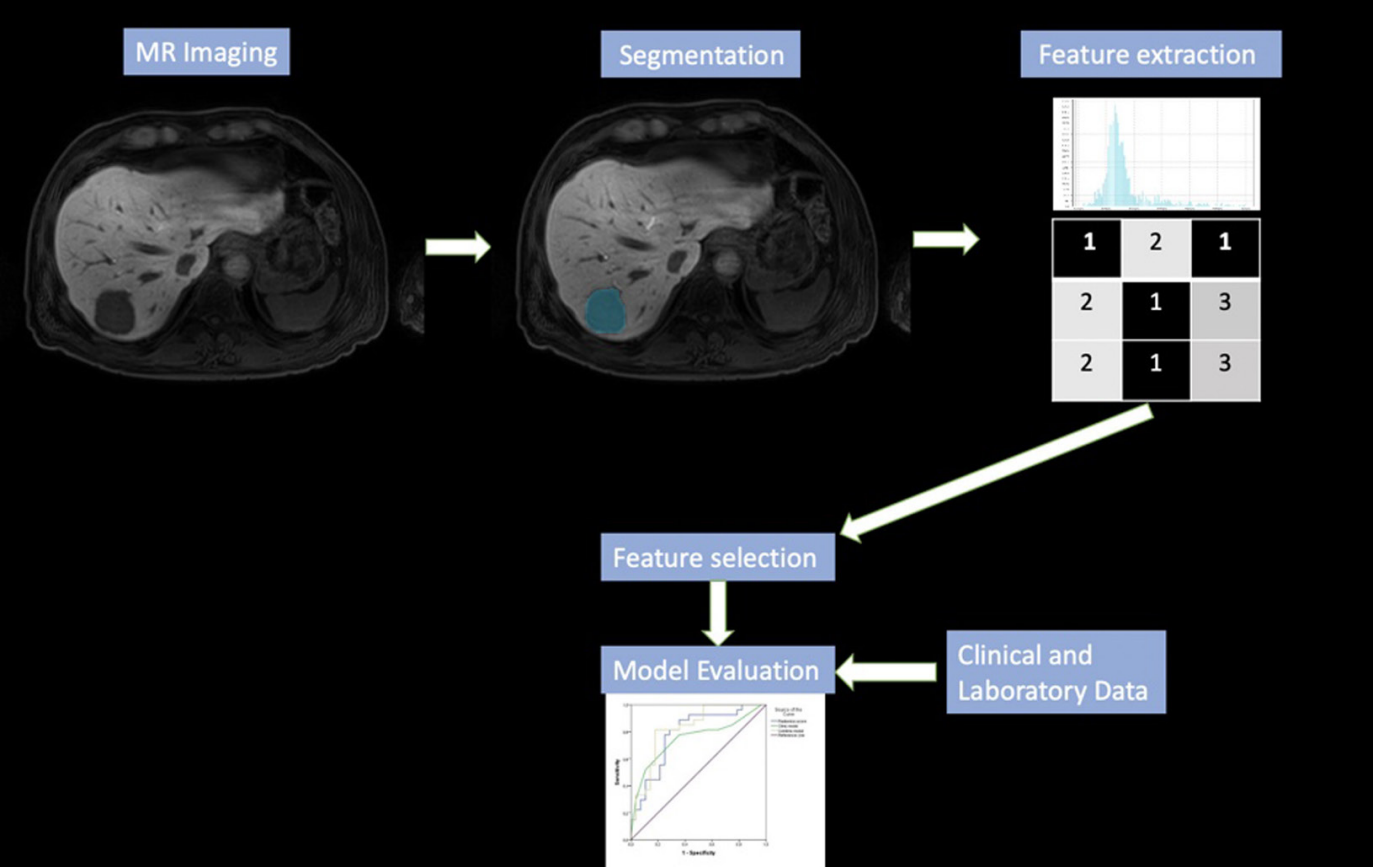
Schematic representation of the radiomics workflow.

The models’ accuracy was assessed using the area under the curve (AUC) after creating receiver operating characteristic (ROC) curves. The model’s predictive power was evaluated using ROC curves and related classification measures, including AUC, sensitivity, specificity, and accuracy. Each workflow of radiomics analysis and model building is shown in Figure 2.

### Statistical Analysis

SPSS software 20.0 (IBM SPSS Corp.; Armonk, NY, USA) and R software (version 1.0.143, https://www.rstudio.com/) (R Foundation for Statistical Computing, Vienna, Austria) were used to conduct the statistical analyses. The chi-square test was first employed to evaluate the relationship or distinction between categorical variables. The Shapiro–Wilk test was then used to examine the distribution of the continuous variables. The Student’s *t*-test and Mann–Whitney *U*-test were then used to identify traits significantly differing between the HCC groups with well and non-well-differentiated grades. The “glmnet” package in R software performed LASSO LR. The AUCs of the models were compared using the Delong test. The goodness of fit of the models was evaluated with the Nagelkerke R-squared. Net reclassification index (NRI) and integrated discrimination improvement (IDI) were performed using the “PredictABLE” package to evaluate the models’ improvement in their discrimination ability. A 2-sided *P* < .05 was considered statistically significant.

## Results

A total of 68 patients (10 females and 58 males, with a mean age of 64.1 ± 10.2 years) were included in the study. The clinicopathological details of 68 HCC patients, allocated into 33 well-differentiated and 35 non-well-differentiated groups, are shown in Table 2. There were no statistically significant differences among the well-differentiated and non-well-differentiated patients regarding gender, age, and clinicopathologic features (all *P* > .05). The performance of the clinical, radiomics, and combined clinical-radiomics (CCR) models was evaluated for each patient. For the prediction of differentiation, the clinical model obtained by applying the LR method revealed that the ALBI score remained an independent risk factor (*P* < .05). The AUC value of the clinical model in predicting differentiation was obtained as 0.749 (95% CI: 0.697-0.909) with a sensitivity of 0.64 and a specificity of 0.79 (95% CI: 0.615-0.883, *P* = .002).

**Figure 3.**
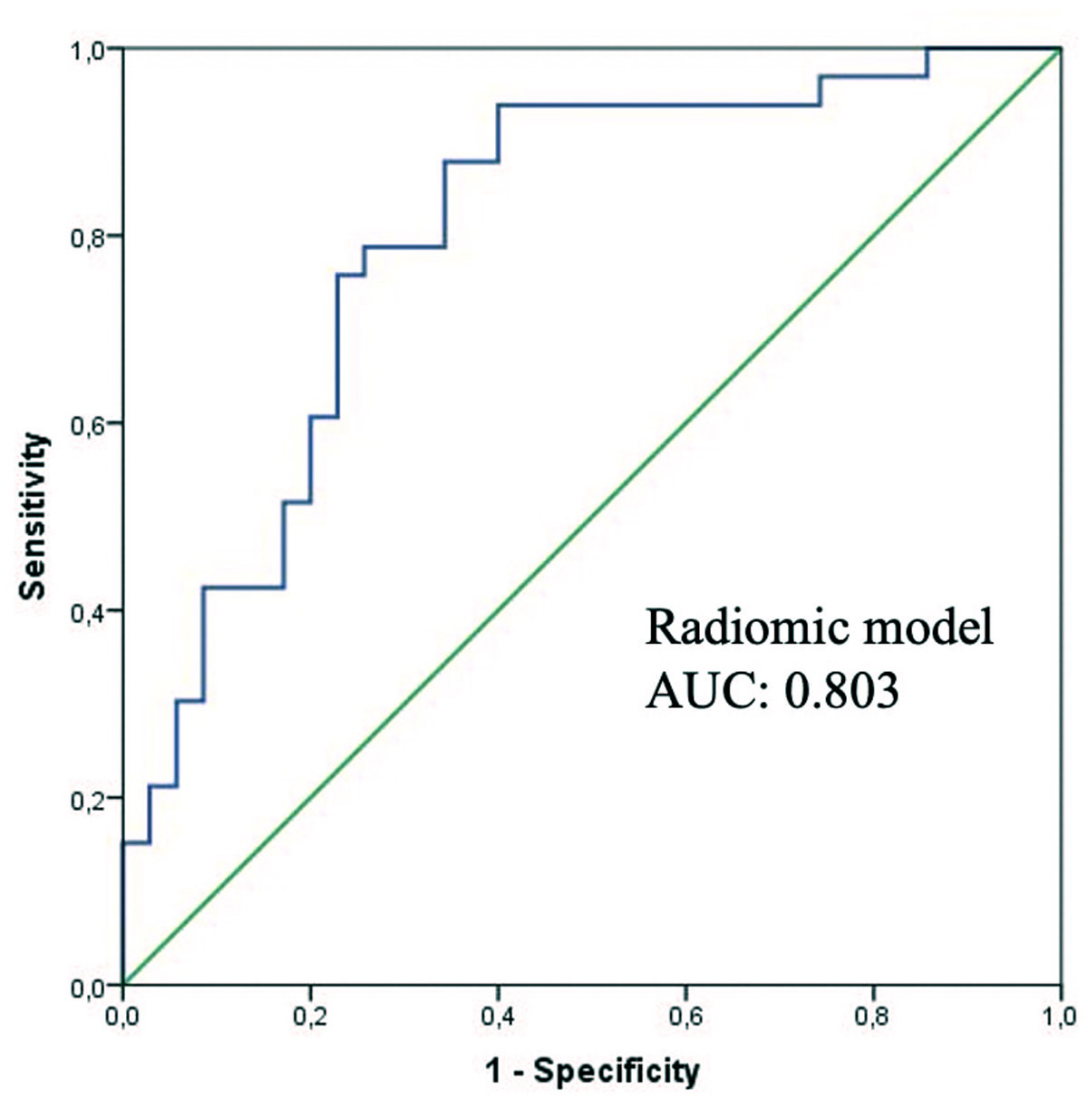
Receiver operating characteristic curve demonstrating the performance of the radiomics model.

**Figure 4.**
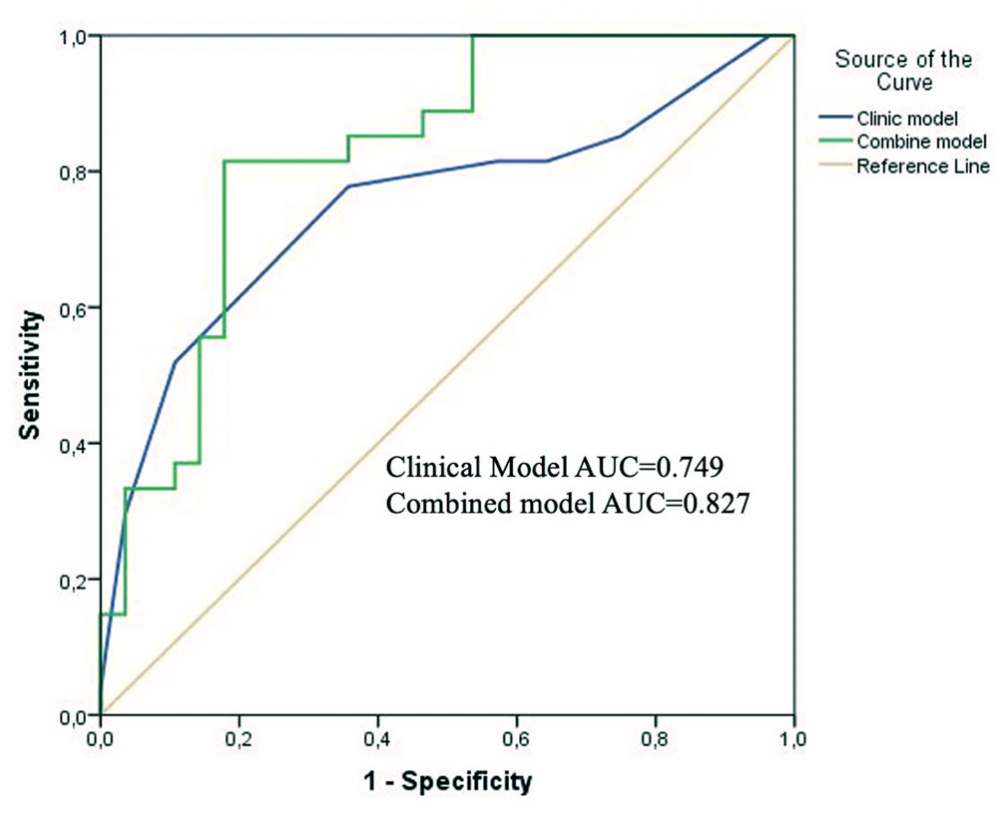
Receiver operating characteristic curves comparing the performance of the clinical model and the combined clinical-radiomics model.

**Figure 5.**
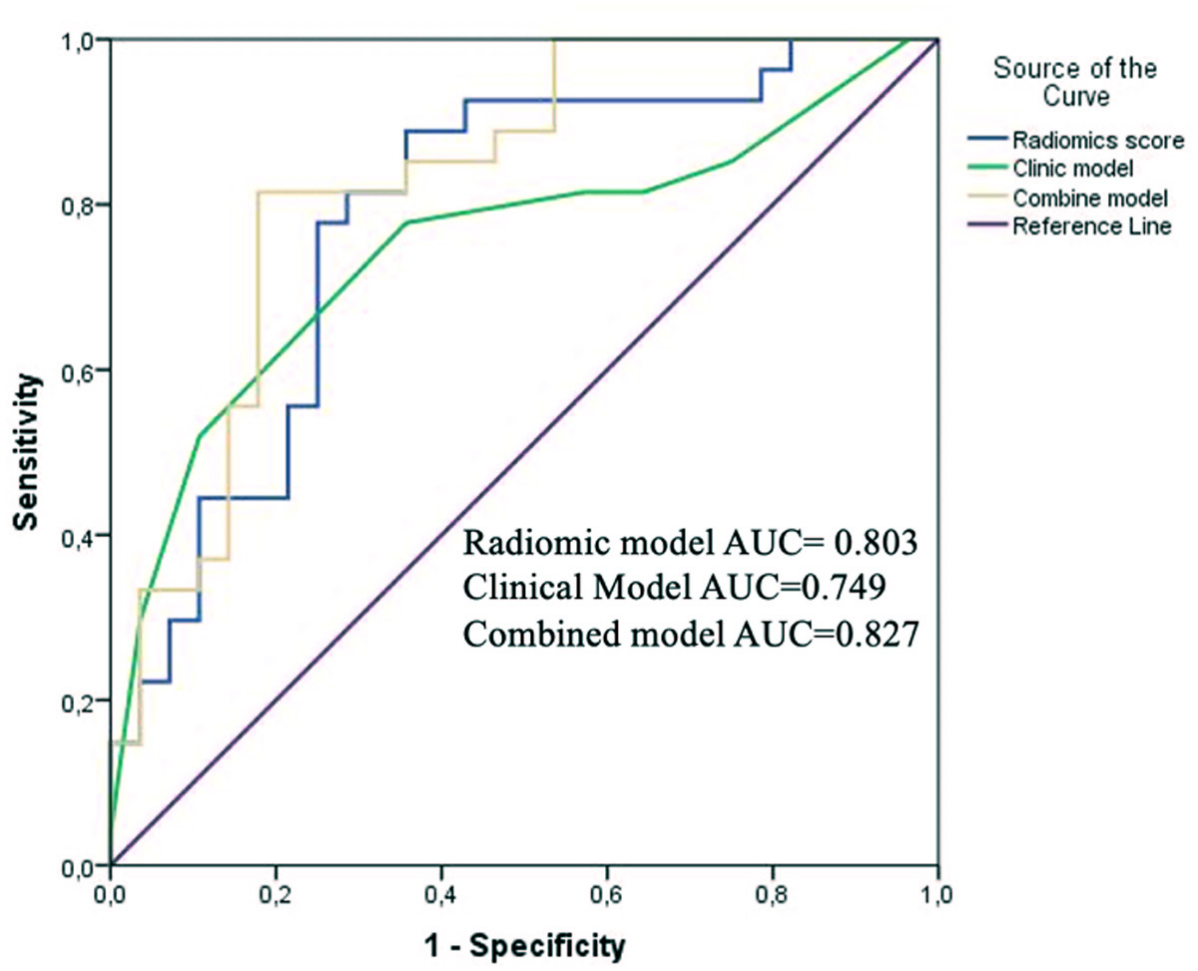
Comparison of receiver operating characteristic curves of all models.

The rad score was significantly lower in the well-differentiated patient group (*P* < .001). The AUC value of the rad score in predicting ES grades was obtained as 0.803 (95% CI: 0.697-0.909) with a sensitivity of 0.77 and a specificity of 0.776 (Figure 3). While the CCR model provided better performance to differentiate ES grades with AUC: 0.827, both sensitivity and specificity are 0.82 (Figure 4) (95% CI: 0.716-0.937, *P* < .001). In the combined model, only radiomics features were a risk factor for differentiation (95% CI: 1.75-23.47, *P* = .005). In addition, it showed no statistically significant improvement over the clinicopathologic model (*P* = .122). Comparison of ROC curves of all models is demonstrated in Figure 5. Compared to the clinical model, the combined model resulted in a 62% (*P* = .015) NRI and 45% (*P* = .003) IDI. Reclassification criteria confirmed that adding the rad score to the clinical model (combined model) performed better than the clinical model. Adding the rad score significantly improved the reclassification performance (Table 3).

## Discussion

This study used radiomics, clinical, and CCR models that utilize gadoxetic acid-enhanced MRI radiomics features and clinical findings to predict HCC histopathological grades. Several studies have proven that radiomics may be helpful in colorectal adenocarcinoma, glioma, bladder cancer, pancreatic neuroendocrine tumors, and soft tissue sarcoma grading.^14^^-^^17^ The histological grade of HCC is crucial for treatment planning, prognosis, and survival after surgical treatment and transplantation.^18^ These results showed that the radiomics model could successfully distinguish HCC tumors into well-differentiated and non-well-differentiated in the dataset. Furthermore, it was proved that non-well-differentiated tumors had significantly higher rad scores (*P* < .001). In addition, the predictive values of the clinical and combined models were evaluated to predict the histological grade of HCC. This study indicated that the ALBI score is an independent risk factor that could distinguish between HCC grades for the first time (*P* < .05). Several studies declared that the ALBI score significantly predicted survival in HCC patients.^19^^,^^20^ This study found that the ALBI score was lower in the well-differentiated group and was identified as an independent risk factor (*P* < .05). This research revealed that the clinical model performed the least well (with an AUC of 0.749). The combined model performed the best in determining the HCC grade, with AUCs of 0.827, and radiomics features were the only independent risk factor that could discriminate between well and non-well-differentiated HCC grades (*P* = .005). Net reclassification index and IDI confirmed that integrating the rad score into the clinical model improves model performance.

Mao et al^21^ developed predictive models based on gadoxetic-acid-enhanced MRI radiomics to evaluate the histological grade of HCC and to analyze the effectiveness of LR and artificial neural network (ANN) models in the AP and hepatobiliary phase (HBP). The AUCs of ANN-HBP and LR-HBP were 0.941 and 0.819 in the test set, whose research methods differ fundamentally from ours.^21^ The aim is to enhance the model’s success by using machine learning algorithms with different and more MRI sequences. Hu et al^22^ used LR, support vector machine, and Adaboost modeling to predict HCC grading using gadoxetic acid-enhanced MRI radiomics features. The AUC of the 3 models was 0.70, 0.67, and 0.61, respectively, in the external test cohort. Moreover, the model’s performance increased with the addition of clinical findings, consistent with this study.^22^ According to Wu et al,^23^ combined models consisted of clinical parameters and T1WI (T1-weighted imaging) and T2WI (T2-weighted imaging) image-based radiomics signatures to discriminate well and poorly differentiated HCCs with AUCs for the test dataset ranging from 0.742 to 0.800, and lower than the AUC of this study. Zhou et al^24^ showed that the texture features extracted from 46 HCC patients’ Gadolinium-DTPA-enhanced magnetic resonance images reflect biologic aggressiveness and predict the histological grading of HCC (with AUCs of 0.827-0.918). According to Mao et al,^25^ the radiomics signatures of contrast-enhanced computed tomography images could predict the pathological grades of HCC with AUC values of 0.731 and 0.718 in the training and test datasets,^25^ respectively, which were the current study’s results. It might be connected to the fact that magnetic resonance images enhanced with Gd-EOB-DTPA provide additional details regarding tumor heterogeneity.

This study has several limitations. First, it was retrospective and included a relatively small number of HCC patients; the findings may not be widely generalizable. Second, a small sample size can reduce the statistical power of the analysis and increase the risk of selection bias. Third, the use of different MRI scanners and scanning parameters could have introduced variability in the extracted imaging features, potentially affecting the model’s performance. While feature normalization was applied to minimize these effects, some degree of bias may still remain. Another important consideration is the cost of hepatocyte-specific contrast agents like gadoxetic acid, which is higher than that of conventional extracellular contrast agents or alternative diagnostic methods. In addition, these models only included specific clinical parameters and hepatobiliary phase MRI radiomics features, while AP and diffusion-weighted imaging may also provide valuable information for HCC grading; these will be considered in future prospective analyses. Furthermore, while all patients underwent pre-treatment Tru-cut biopsy, surgical specimens were not used as the primary histopathological reference to avoid potential verification bias. Nevertheless, biopsy-based grading is inherently limited by sampling error, as only a portion of the tumor is assessed, whereas radiomics evaluates the lesion as a whole. This discrepancy represents an important methodological constraint. Finally, it is acknowledged that a small subset of moderately differentiated HCCs may demonstrate increased OATP (organic anion transporting polypeptide) expression due to genetic alterations. By binarizing the histological grades into well- versus non-well-differentiated groups, this biologically distinct subset may have been overlooked; future subgroup analyses will address this issue more explicitly. In the future, radiomics models with more sequences of MRI will be applied. Finally, differentiation was categorized as well-differentiated and poorly differentiated rather than using ES grades 1, 2, 3, and 4.

A CCR model was developed to predict the histopathological grade of HCC by integrating preoperative gadoxetic-acid-enhanced hepatobiliary phase MRI radiomics features with clinical parameters. The model effectively distinguished between well-differentiated and non-well-differentiated tumors. Future multicenter studies with larger cohorts and external validation are essential to confirm these findings before clinical application.

## Figures and Tables

**Table 1.** Radiomics Features Selected by Least Absolute Shrinkage and Selection Operator

Features	Coefficient
Original shape flatness	−0.7333704
Original first order minimum	−0.3624622
Original first order 10th percentile	−0.3264919
Original gray level co-occurrence matrix cluster prominence	−5.697986e-07
Original gray level co-occurrence matrix cluster shade	−4.175423e-05
Original gray level co-occurrence matrix maximal correlation coefficient	−9.626540
Original neighboring gray tone difference matrix busyness	0.05846716

**Table 2.** The Clinicolaboratory Data of Individuals with Hepatocellular Carcinoma

	Differentiation		*P*
	Non-Well (n = 35)	Well (n = 33)	
Age (years)	62.8 ± 9.3	65.4 ± 10.9	.235
Gender, n (%) Male Female	31 (88.6) 4 (11.4)	27 (81.8) 6 (18.2)	.507
AFP, n (%) <200 ng/mL >200 ng/mL	25 (73.5) 9 (26.5)	23 (69.7) 10 (30.3)	.939
Hepatitis infection, n (%) Negative Hepatitis B Hepatitis C	13 (46.4) 11 (39.3) 4 (14.3)	5 (18.5) 15 (55.6) 7 (25.9)	.083
Portal vein thrombosis, n (%) Without With	25 (71.4) 10 (28.6)	25 (75.8) 8 (24.2)	.897
Ascites, n (%) Without With	29 (82.9) 6 (17.1)	32 (97.0) 1 (3.0)	.107
Tumor size, n (%) <5 cm >5 cm	14 (40.0) 21 (60.0)	10 (30.3) 23 (69.7)	.560
Tumor type, n (%) Nodular Infiltrative	29 (82.9) 6 (17.1)	26 (78.8) 7 (21.2)	.906
Portal hypertension n (%) Without With	26 (74.3) 9 (25.7)	24 (72.7) 9 (27.3)	>.999
Tumor distribution, n (%) Unilobar Bilobar	23 (65.7) 12 (34.3)	24 (72.7) 9 (27.3)	.717
ALBI score, n (%) 1 2 3	11 (31.4) 18 (51.4) 6 (17.1)	11 (33.3) 20 (60.6) 2 (6.1)	.359
CP grade, n (%) A B	27 (77.1) 8 (22.9)	26 (78.8) 7 (21.2)	>.999
Total bilirubin	0.9 (0.3-11.0)	1.0 (0.5-3.5)	.521
NLR	2.3 (0.6-13.6)	2.6 (1.1-11.3)	.778
ALT	29.0 (9.0-75.0)	39.0 (10.0-671.0)	.118
INR	1.1 (0.9-1.9)	1.1 (0.9-1.5)	.761

AFP, alpha-fetoprotei; ALBI, albumin-bilirubin; ALT, alanine aminotransferase; CP, Child–Pugh; INR, international normalized ratio; NLR, Neutrophil-to-lymphocyte ratio.

**Table 3.** Evaluation of Model Performances

	Cutoff	Sensitivity	Specificity	AUC (95% CI)	*P*	NRI (95% CI)	p	IDI (95% CI)	*P*
Clinical model	0.517418	0.64	0.79	0.749 (0.615-0.883)	.002				
Combined model	0.389291	0.82	0.82	0.827 (0.716-0.937)	<.001	0.62 (0.12-1.12)	0.015	0.45 (0.05-0.24)	.003

Nagelkerke *R*^2^ is 0.258 for the clinical model and 0.446 for the combine model.

AUC, area under the curve; IDI, integrated discrimination improvement; NRI, net reclassification index.

## Data Availability

The data that support the findings of this study are available on request from the corresponding author.
